# Population Collapse of the Spidery Wattle (*Acacia araneosa*) During Late Holocene Aridification: Genomic Evidence for Critically Endangered Status

**DOI:** 10.1002/ece3.73959

**Published:** 2026-07-10

**Authors:** Brittany P. Hogben, Andrew J. Lowe, Chelsea Hampel, Lars Littmann, Martin O'Leary, James B. Dorey, Colette Blyth

**Affiliations:** ^1^ School of Biological Sciences Adelaide University Adelaide South Australia Australia; ^2^ Environment Institute Adelaide University Adelaide South Australia Australia; ^3^ Department of Zoology, Centre for Palaeogenetics Stockholm University Stockholm Sweden; ^4^ State Herbarium of South Australia Adelaide South Australia Australia; ^5^ Environmental Futures Research Centre, School of Science University of Wollongong Wollongong New South Wales Australia; ^6^ Molecular Horizons Research Institute, School of Science University of Wollongong Wollongong New South Wales Australia; ^7^ Institute of Silviculture University of Natural Resources and Life Sciences (BOKU) Vienna Austria

**Keywords:** climate change, conservation genetics, Fabaceae, past demography, Stairway Plot, threatened species

## Abstract

Protection and restoration of ecosystems, ceasing human‐induced extinctions and maintaining genetic diversity are key goals for the Global Biodiversity Framework. Molecular techniques can provide empirical data to understand all these goals and provide actionable conservation advice. *Acacia araneosa*, an endangered aridland shrub, has a restricted distribution in South Australia where it coexists sympatrically with the widespread *Acacia rivalis* . Evidence of hybridisation between the two challenges the *A. araneosa* species concept. Here, we examine the genetic distinctness between the two species and use a genomic approach to estimate population genetic structure and historical population size. We used SNP data to quantify species boundaries, population structure, demography, kinship and genetic diversity within and between populations of both *A. araneosa* and *A. rivalis* . We found that while *A*. *araneosa* and *A. rivalis* hybridise to produce F1 hybrids, further backcrossing and gene flow are limited, suggesting that a post‐zygotic breeding barrier may exist and that the two species are distinct. Genetic structure within *A. araneosa* suggests limited gene flow between the two remaining populations occurring within its small range. Our results also reveal a major reduction in effective population size (*Ne*) in recent times, dropping from ~49,000 to ~51 (−99.9%) over the last 3200 years, coinciding with widespread climatic drying across southern Australia. Paleoclimatic data over the last 3000 years in arid Australia are uncertain; hence, we provide novel empirical support for continued drying in this region using molecular techniques. We recommend that *A. araneosa* be listed as Critically Endangered on the IUCN Red List, and that the remaining populations are actively conserved and managed to maintain (and ideally increase) population size and genetic diversity.

## Graphical abstract

*Acacia araneosa* is an extremely range‐restricted aridland shrub endemic to South Australia, coexisting sympatrically with the more common *Acacia rivalis* . Using SNP data we explore the species boundaries between the two and find evidence of non‐threatening hybridisation. We find strong and short‐range barriers to gene flow between the two *A. araneosa* populations, and evidence of major declines in population size, supporting a Critically Endangered listing for the species.

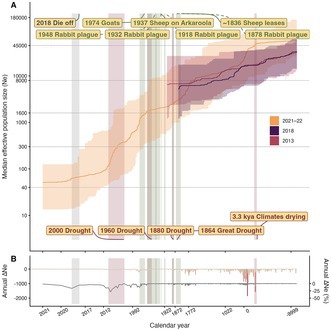

## 1 Introduction

Globally, we are facing a biodiversity crisis. In Australia, an estimated 1490 plant species are Critically Endangered, Endangered or Vulnerable (Department of Climate Change‚ Energy‚ the Environment and Water 2025), and approximately 35 plant species have become extinct, primarily due to habitat loss (Woinarski et al. 2019). Besides requiring suitable habitat, plant species need to be resilient to climate change and anthropogenic pressures to avoid extinction. Loss of genetic diversity within a species reduces its adaptability to change and, in the case of threatened plant species, this loss of diversity can be accelerated by already reduced population sizes (Ottewell et al. 2016). Smaller, more isolated populations are subject to processes which have a negative impact on genetic diversity, such as fixation of maladaptive traits and inbreeding depression (Blyth et al. 2020).

Baseline population size estimates are lacking for most species and are often considered in the context of contemporary, rather than ancient, anthropogenic impacts (Rodrigues et al. 2019). However, species extinctions have continued throughout the Holocene (Turvey 2009) and so Holocene declines, that haven't caused extinctions, are likely widespread. Ancient declines are also expected to impact contemporary extinction risks (Costion et al. 2012) and might already have put species on a trajectory towards an extinction vortex. Hence, where possible, ancient declines should be inferred and used to contextualise and understand contemporary extinction risks. Indeed, ancient population declines are detectable using genetic data and demographic reconstructions and can inform conservation and be used to test hypotheses of anthropogenically and environmentally driven changes (Dorey et al. 2021).

For many taxa, conservation is further complicated by uncertain species boundaries. Accurate species classification and confirmation of genetic isolation is an essential first step in achieving positive conservation outcomes (Ottewell et al. 2016). Since threatened species lists are heavily relied upon to guide resource allocation (Burgman et al. 2007), misidentification can lead to misplaced conservation priorities, diverting resources away from species in need and towards more common taxa. Genetic techniques have long been used in conservation studies to complement and enhance morphological trait data (Chambers and MacAvoy 1999).

Difficulties in recognising distinct taxa are further complicated by the occurrence of hybridisation between closely related species. Not only is hybridisation an impediment to management, it can also be a major threat for threatened species, particularly those that exist sympatrically with more abundant species (Allendorf et al. 2001; Rutherford et al. 2019). Hybridisation has the potential to increase genetic diversity within populations; however, hybrids do not always backcross with their parental species and are not always fertile, essentially removing them from the gene pool of one or both parent species while remaining as competitors (Abbott and Brennan 2014). Failure to recognise hybridisation between closely related species can result in misconceptions regarding the genetic health of a population (Binks et al. 2015), or lead to increased risk of inbreeding (Edmands 2007). Continuous hybridisation might result in outbreeding depression, either intrinsic, defined as the break‐up of co‐adapted gene complexes (Whitlock et al. 2013), or extrinsic, reduced adaptation to environmental conditions (Allendorf et al. 2001). In extreme circumstances, hybridisation can even contribute to the extinction of taxa through replacement and genetic mixing (Allendorf et al. 2001; Prentis et al. 2007; Todesco et al. 2016).

The increasing accessibility and use of molecular techniques in conservation science provides major opportunities to understand population dynamics. We should expect population dynamics to be linked with environmental changes, both past and future (Pacifici et al. 2015). Molecular techniques can also be used to investigate the deep past and make inferences about environmental changes that are currently impossible—at least in some regions—using paleoclimatic data. However, such inferences require in‐depth understanding of the biology of a taxon, the history of habitat change within its range, and interdisciplinary research. One taxon for which much of the biology, regional history and interdisciplinary data already exist is *Acacia araneosa* Whibley (Spidery Wattle; Fabaceae). Genomic techniques have never been applied to *A. araneosa*, leaving major and foundational knowledge gaps for its conservation and major opportunities to learn about both the species and regions of arid Australia.

The taxonomic validity of *A. araneosa*, a South Australian endemic aridland shrub species of conservation concern, has been called into question due to phenotypic plasticity and possible hybridisation with congenerics. Presently listed as Vulnerable under the national Environment Protection and Biodiversity Conservation (EPBC) Act 1999 and Endangered under the National Parks and Wildlife Act 1972 (South Australia), *A. araneosa* is thought to hybridise with the broadly distributed and closely related *Acacia rivalis* J.M.Black (Silver Wattle; Fabaceae) (Whibley et al. 1985) with other threats including browsing by feral animals in the region, increasingly steadily from around the 1840s (including sheep, goats, rabbits, camels and donkeys), which both reduce seedling recruitment and increase adult mortality rates (Davies 2000).

Currently restricted to the Arkaroola Wilderness Sanctuary and its shared border with Vulkathunha‐Gammon Ranges National Park, *A. araneosa* is range‐restricted, with a small population size and limited distribution. Across two sites, the species is typically found on hill slopes and has been strongly associated with calcareous soils overlying dolomitic substrate. *Acacia araneosa* is believed to be a relic of an era with a more temperate climate, surviving in two refuge subpopulations of 8 and 5 km^2^ which are separated by a ridge and a valley (Department for Environment and Heritage 2006; South Australian Arid Lands Natural Resources Management Board 2010). Landholders have observed population declines, low recruitment, adult tree mortality and susceptibility to grazing pressures from feral animals (Davies 1995). These trends have persisted despite the installation of experimental feral animal exclusion fences, and subsequent declines have been attributed to drought (South Australian Arid Lands Natural Resources Management Board 2010).

**FIGURE 1:**
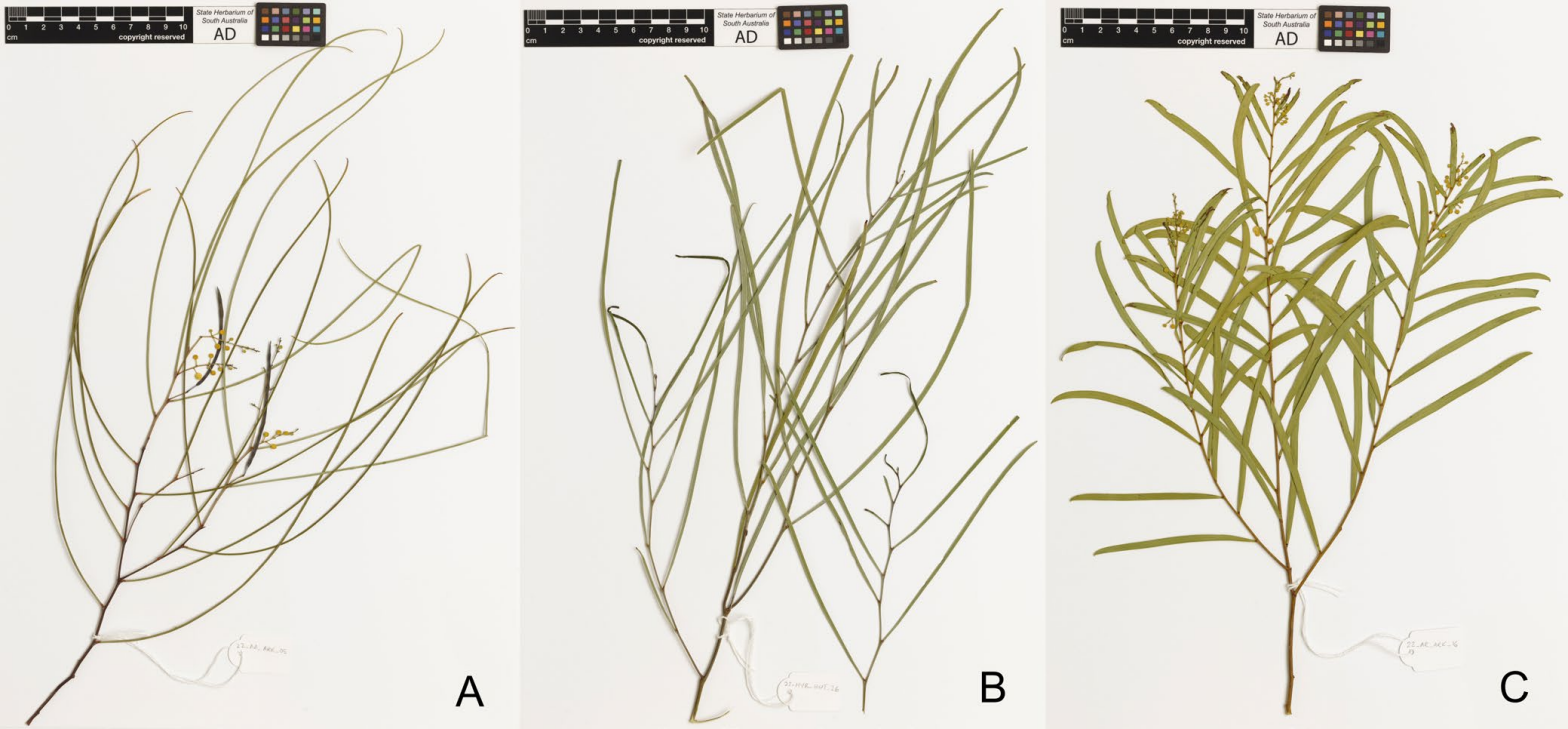
Specimens collected for each distinct phenotype. (A) *Acacia araneosa*, (B) putative hybrids and (C) *Acacia rivalis* .

When germinated and cultivated outside of its native range, seeds from *A. araneosa* have been informally reported to develop into a form resembling *A. rivalis* (South Australian Arid Lands Natural Resources Management Board 2010). The phyllodes of *A. rivalis* are flat, linear and approximately 2–5 mm wide—a contrast to the filiform phyllodes of *A. araneosa* (Figure 1). This apparent phenotypic plasticity, along with an observed intermediate phenotype between the two species, raised questions about the validity of the species concept and current conservation listing, both of which have relied on morphology. With excellent background information on *A. araneosa* and the contemporary history of the region, we have a valuable opportunity to address major hypotheses about species boundaries, environmental change and species conservation using genomic data.

We have two over‐arching hypotheses to inform the conservation management actions for *A*. *araneosa—*to guide practical conservation efforts—and to better understand contemporary and ancient environmental changes in arid southern Australia. We hypothesise that *A. araneosa* and *A. rivalis* are distinct species, with limited or no contemporary gene flow. We will compare measurable levels of genetic diversity, degree of inbreeding and within‐species kinship between *A. araneosa*, *A. rivalis* and any putative hybrid phenotypes between the two species. Since contemporary and ancient declines in *A. araneosa* are suspected, we hypothesise that declines in effective population size (Ne) can be detected using genetic data. Using genomic data, we will examine population structure and changes in effective population size through time. This will inform conservation assessments for *A. araneosa* and hopefully provide context of contemporary and ancient environmental changes that are, for arid southern Australia, otherwise lacking.

## 2 Materials and Methods

### 2.1 Study Species and Sample Collection

**FIGURE 2:**
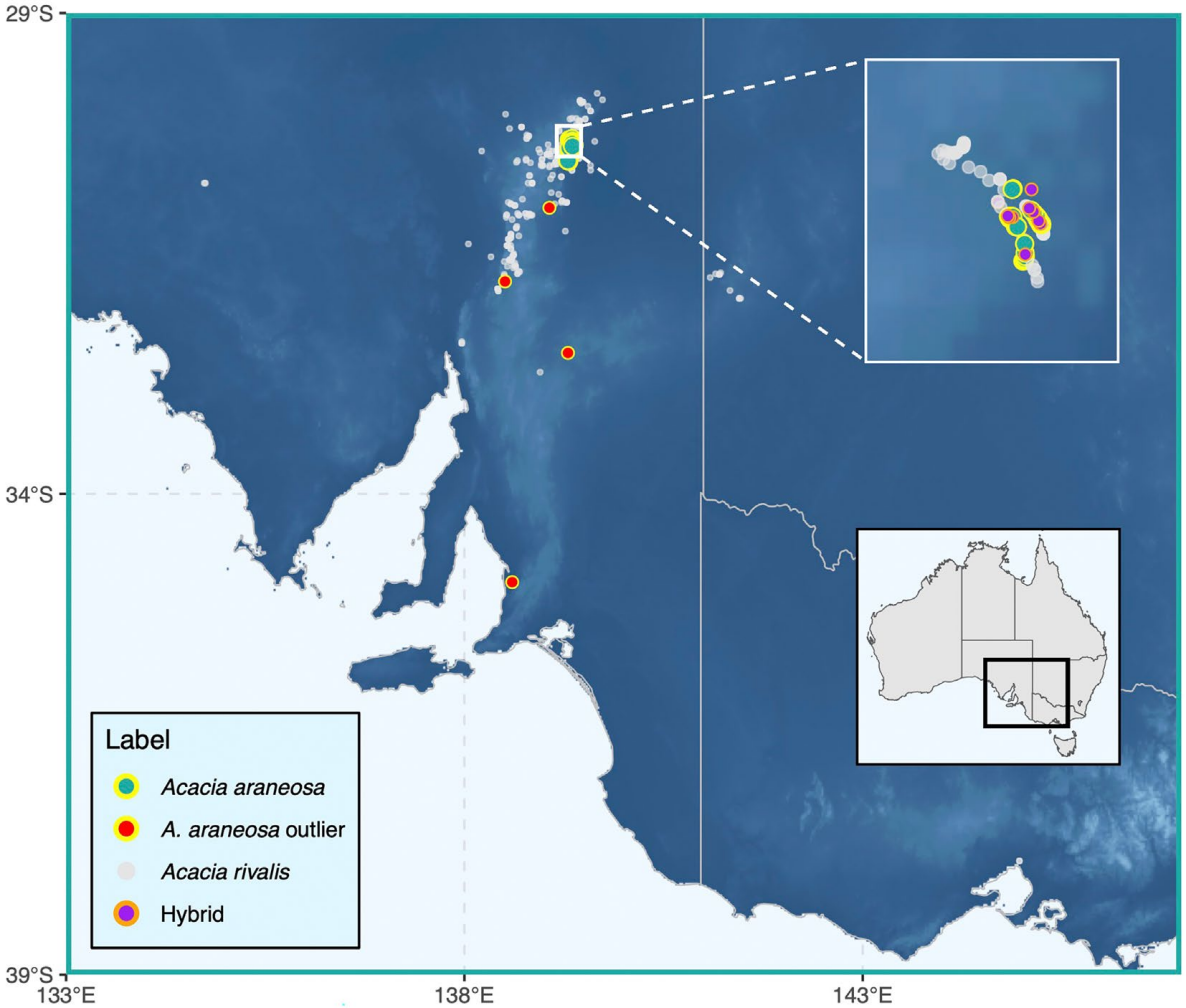
The *Acacia araneosa* points (green) and expert‐identified outliers (red) along with *Acacia rivalis* (grey) points from the Atlas of Living Australia, downloaded using galah version 2.1.2 (Stevenson et al. 2022).

An initial sampling trip in November 2021 aimed to make range‐wide collections of *A*. *araneosa* across the two known localities within the Arkaroola Wilderness Sanctuary, as well as collecting *A. rivalis* individuals near the *A. araneosa* subpopulations, and any hybrids/intermediate phenotypes between the two species (Figures 1 and 2). Where enough individuals were present, sampling skipped nearest neighbours to avoid closely related individuals (i.e., to minimise sampling of clonal or sibling pairs) but all individuals presumed to be hybrids were sampled. Samples were taken from the uniform canopy height of the plant when possible, and approximately 5–10 young leaves were collected from each sample of interest. These leaves were stored in labelled gauze tea bags and placed into secure containers with silica beads in the field for later DNA extraction (Chase and Hills 1991). In addition, ten *A. araneosa* leaf tissue samples from Terrestrial Ecosystem Network (TERN) AusPlot sampling trips in 2013 (*n* = 5) and 2018 (*n* = 5) were included in the study.

A second trip was conducted in 2022 to increase the sample size, following up on personal communications with landholders who had observed some recruitment of *A. araneosa* and an unusual phenotype of *A. rivalis* near the Nooldoonooldoona waterhole (Doug Sprigg, pers. comm., 2022). We collected specimens representative of all phenotypes found for all species and putative hybrids. We lodged these samples at the State Herbarium of South Australia and confirmed our identifications with *Acacia* taxonomist, Martin O'Leary. Our final sample dataset after expert identification consisted of 95 *A. araneosa* samples, 71 *A. rivalis* samples and 17 putative hybrids.

### 2.2 DNA Extraction and Sequencing

DNA extraction was undertaken at the Advanced DNA, Identification and Forensic Facility (ADIFF), University of Adelaide. Library preparation and sequencing were performed at the Australian Genome Research Facility (AGRF). For DNA extraction, the Machery‐Nagel Nucleospin Plant II Kit and Qiagen DNeasy Plant Mini Kit were used following standard protocols. To obtain a reduced representation of the genome for SNP discovery across all samples, double‐digest restriction‐associated DNA sequencing (ddRADseq) was applied. Detailed descriptions of the method can be found in Peterson et al. (2012). In brief, the ddRADseq protocol included the following steps: (1) digestion of genomic DNA using two restriction enzymes, PstI and MseI; (2) ligation of barcoded adaptors to the restriction overhangs specific to each sample; (3) size selection of pooled fragments within the range of 280–342 bp using the Blue Pippin system (Sage Science, Beverly, MA, USA); and (4) library amplification through 11 cycles of polymerase chain reaction (PCR) using indexed primers. Libraries were evaluated via gel electrophoresis (Agilent D1000 ScreenTape Assay), quantified using quantitative PCR (qPCR) with KAPA Library Quantification Kits for Illumina, and sequenced on the NextSeq 500 system with 150 bp single reads, using the NextSeq 500 High Output Kit v2 (150 cycles).

Post‐sequencing, reads were processed through the STACKS pipeline (Catchen et al. 2013) at AGRF (Melbourne, Australia) and assembled using the reference genome of the Australian wattle species, *Acacia pycnantha* Benth. (McLay et al. 2022). Initially, reads were demultiplexed using inline barcodes, checked for quality and assessed for the presence of restriction sites, resulting in individual FASTQ files per sample. Reads were then trimmed to a uniform length (shortest read minus 2 bp) to account for variations in barcode lengths. For each sample, stacks of similar reads referred to as tags were generated. Tags common across all samples were compiled into a catalogue, variants were called and genotypes assigned to these polymorphic loci.

### 2.3 Data Analysis

#### 2.3.1 Filtering Results

We read the VCF file into R version 4.4.2 (R Development Core Team 2019) and RStudio version 2024.12.0+467 (RStudio Team 2020). We used the package vcfR version 1.15.0 (Knaus and Grunwald 2017) to import the VCF and extract the read depth data but used a custom function, depthFun (https://github.com/jbdorey/Acacia_SNPs), to filter for minimum and maximum read depth. We used a minimum read depth of 5 and a maximum read depth of $d+4\sqrt{d}$, where *d* = the mean depth according to (Li 2014). We then used vcfR to export the modified VCF file to be read in by the package dartR.base version 0.98 (Mijangos et al. 2022) as a genlight file in R. We modified the genlight metadata and data, along with other aspects of our code, using packages in the tidyverse version 2.0.0 (Wickham et al. 2019).

We filtered our genlight files using a custom filtering function, filteringFunction (https://github.com/jbdorey/Acacia_SNPs), to wrap dartR.base functions and ensure their consistent application. We filtered for ‘all NA’, monomorphic loci, locus call rate (threshold = 0.9), individual call rate (threshold = 0.8), minor allele frequency (MAF = 0.05) and linkage disequilibrium (LD R‐squared = 0.2). We applied these filters across four datasets for various analyses—(a) all specimens including the outgroup, (b) all specimens except for the outgroup, (c) only *A. araneosa* and *A. rivalis* —that is, without any hybrids, and (d) *A. araneosa* only.

#### 2.3.2 PCoA

We used dartR.base (Mijangos et al. 2022) to conduct a principal coordinate analysis on the dataset (b; all specimens except for the outgroup) then plotted the results using ggplot2 version 3.5.1 (Wickham 2016) to visualise dissimilarity between the populations based on a genetic distance matrix.

#### 2.3.3 Tree Building

We calculated Euclidean genetic distance by individual, on (b; all specimens except for the outgroup), using dartR.base. We used the package StAMPP (Pembleton et al. 2013) to export the distance matrix as a phylip file, which was then imported into SplitsTree version 6.3.41 (Huson and Bryant 2024) to create a phylogenetic tree.

#### 2.3.4 Isolation by Distance

To explore the relationship between geographic and genetic distance, we used the package dartR.spatial version 0.78 (Mijangos et al. 2022) to perform an isolation by distance (IBD) analysis on the dataset (d; *A. araneosa* only) calculated using Euclidean distance.

#### 2.3.5 Tess3

Using the package Tess3 version 1.1.0 (Caye et al. 2016), we estimated spatial population structure and ancestry coefficients for (d; *A. araneosa* only). We calculated ancestry coefficients using an alternating projected least squares algorithm for *K* 1–5, with ten repetitions per *K*‐value. After assessing the cross‐validation scores, *K* = 2 was had most support, and was selected to generate an ancestry coefficient *Q*‐matrix. We used the plot function in Tess3 to interpolate the values of the *Q*‐matrix onto a map using the Fields Krig Model with a theta value of 10.

#### 2.3.6 Effective Population Size and Demographic Reconstruction

To estimate contemporary effective population sizes (*Ne*), we used dartR.popgen version 1.0.0 (Mijangos et al. 2022) to call NeEstimator version 2 (Do et al. 2014) from within R. We estimated *Ne* for both *A. araneosa* and *A. rivalis* and used critical values, minor allele frequencies, 0.00 and 0.05. To estimate past demography we used dartR.popgen to call Stairway Plot 2 version 2.1.1 (Liu and Fu 2020). We used a tag length of 300 (multiplied by the number of loci), a mutation rate of 6.95 × 10^−9^ from *Arabidopsis thaliana* (Weng et al. 2019), 200 bootstrap replicates, a generation time of 2 years, and 67% of SNPs used for training. For both NeEstimator and Stairway Plot, we analysed datasets for the collection years 2013, 2018 and 2021 + 2022 (combined as these samples were collected within a two‐year generation time) individually.

#### 2.3.7 Kinship

Pairwise kinship coefficients across all *A. araneosa*, *A. rivalis* and hybrid samples were calculated using the KING‐robust estimator implemented in PLINK 2 (Chang et al. 2015; Manichaikul et al. 2010). In this approach, kinship coefficients are scaled such that duplicate samples or monozygotic twins have expected values of approximately 0.5, first‐degree relatives (parent‐offspring or full siblings) have expected values of ~0.25, and second‐degree relatives (aunt, uncle, grandparent, grandchild, niece, nephew or half‐sibling) have expected values of ~0.125. Following the recommendations of Chang et al. (2015), thresholds were set using the geometric mean between categories: pairs with kinship ≥ 0.354 were identified as duplicate or monozygotic twin pairs and excluded; ≥ 0.177 and < 0.354 were classified as first‐degree relationships; ≥ 0.0884 and < 0.177 as second‐degree relatives; and < 0.0884 as more distant or unrelated.

As a quality control step, the dataset included technical duplicate samples, which clustered in the ‘duplicate/monozygotic twin’ category (kinship ≥ 0.354), confirming the reliability of the data and correct sample matching. These duplicates were excluded from downstream analyses by applying a kinship threshold of < 0.354.

All data processing and visualisation were conducted in RStudio (2024.12.1+563) using R version 4.4.3. The package dplyr version 1.1.4 (Wickham et al. 2025) was used to assign each pairwise comparison to a kinship category based on the thresholds outlined above, and to summarise the number and proportion of relationships per group pairing, along with the range and mean of kinship coefficients.

#### 2.3.8 Diversity Statistics

We then calculated diversity statistics by locus for the dataset (b; all specimens except for the outgroup) using dartR.base. This included observed heterozygosity (Ho), expected heterozygosity (He), Unbiased expected heterozygosity (uHe) and inbreeding coefficient (*F*_IS_). We also calculated Pairwise *F*_ST_ between *A. araneosa* and *A. rivalis* and between the tess3r‐determined populations within *A. araneosa*, using 1000 bootstrap replicates.

#### 2.3.9 Extent of Occurrence and Area of Occupancy

We extracted *A. araneosa* and *A. rivalis* occurrence records from the Atlas of living Australia using the R packages galah version 2.1.2 (Stevenson et al. 2022) and BeeBDC version 1.3.0 (Dorey, Fischer, et al. 2023; Dorey, O'Reilly, et al. 2023). We used these data, and our collection data, with red version 1.6.3 (Cardoso and Banco 2025) to calculate extent of occurrence (EOO) and area of occurrence (AOO).

#### 2.3.10 IUCN Redlist Assessment

An Internation Union for Conservation of Nature (IUCN) Redlist assessment for *A. araneosa* was conducted following the IUCN Red List categories and criteria, version 3.1 (IUCN 2012).

## 3 Results

### 3.1 Filtering Results

After filtering, dataset a; all specimens, including the outgroup, had 5918 SNPs for 179 specimens with 0.99% missing data. Dataset b; all specimens except for the outgroup had 13,044 SNPs for 165 specimens and had 2.36% missing data. Dataset c; only *A. araneosa* and *A. rivalis* had 3474 SNPs for 149 specimens, with 2.37% missing data. Finally, dataset d; *A. araneosa* only had 1314 SNPs across 84 individuals and 2.08% missing data.

### 3.2 PCoA

**FIGURE 3:**
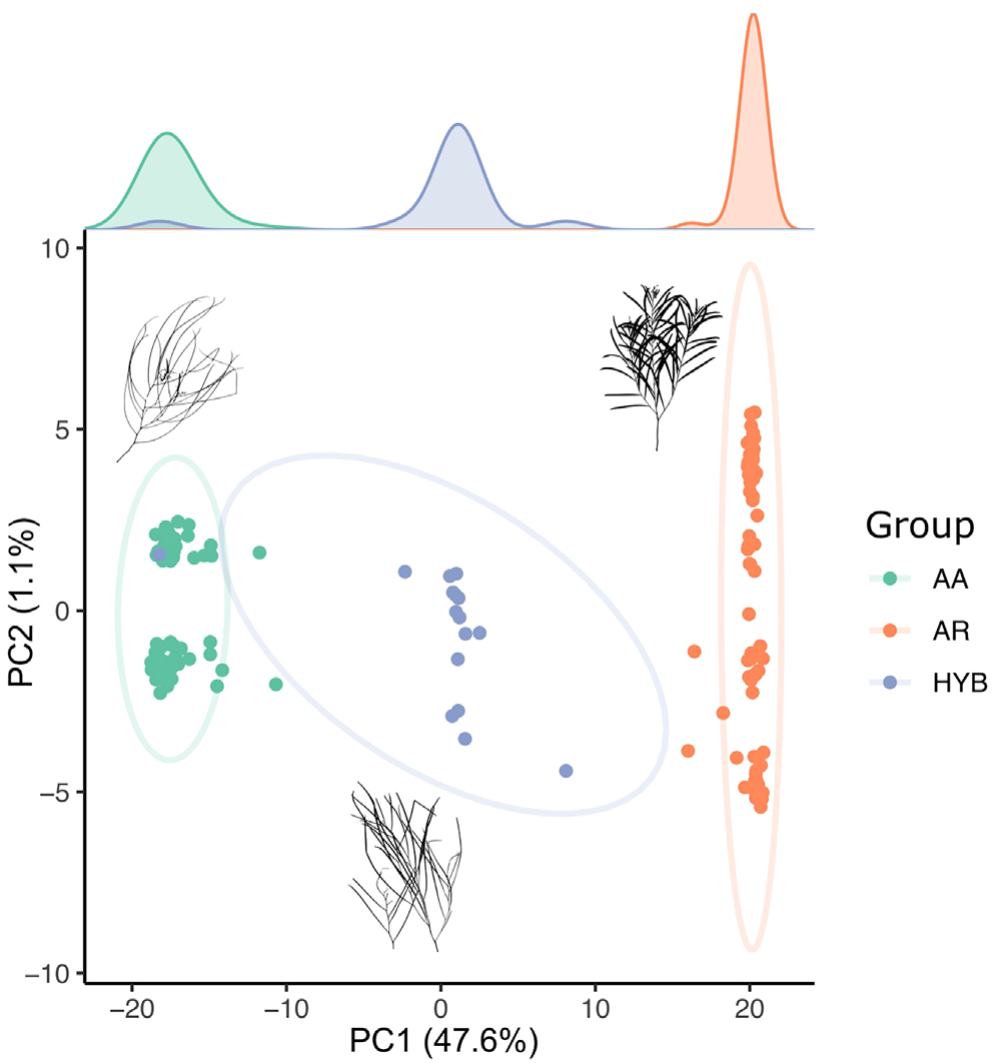
Principal coordinates analysis (PCoA) of genetic distance among samples in the (b; all specimens except for the outgroup) genlight. Silhouettes of branchlets demonstrate the typical phenotype of each group. Points are coloured by group assignment based on the phenotypic morphology of each sample. Axis 1 explained 47.6% of the variation and Axis 2 explained 1.1%. Density plots above the *x*‐axis shows the smoothed frequency of *x*‐axis points (PC1).

Results from the principal coordinates analysis (PCoA) revealed distinct clustering of populations *A. araneosa*, *A. rivalis* and putative hybrids (Figure 3). One putative hybrid (HYB_CAM_01) consistently clustered with *A. araneosa* (rather than with the other hybrids), suggesting it was either misidentified in the field or the result of a lab error and is a pure *A. araneosa* individual. Most variation in our PCoA is explained along the first principal coordinate (47.6%), and the amount of variation explained by the following principal coordinates quickly diminishes, with the second principal coordinate explaining just 1.1% of the variation.

### 3.3 Tree Building

**FIGURE 4:**
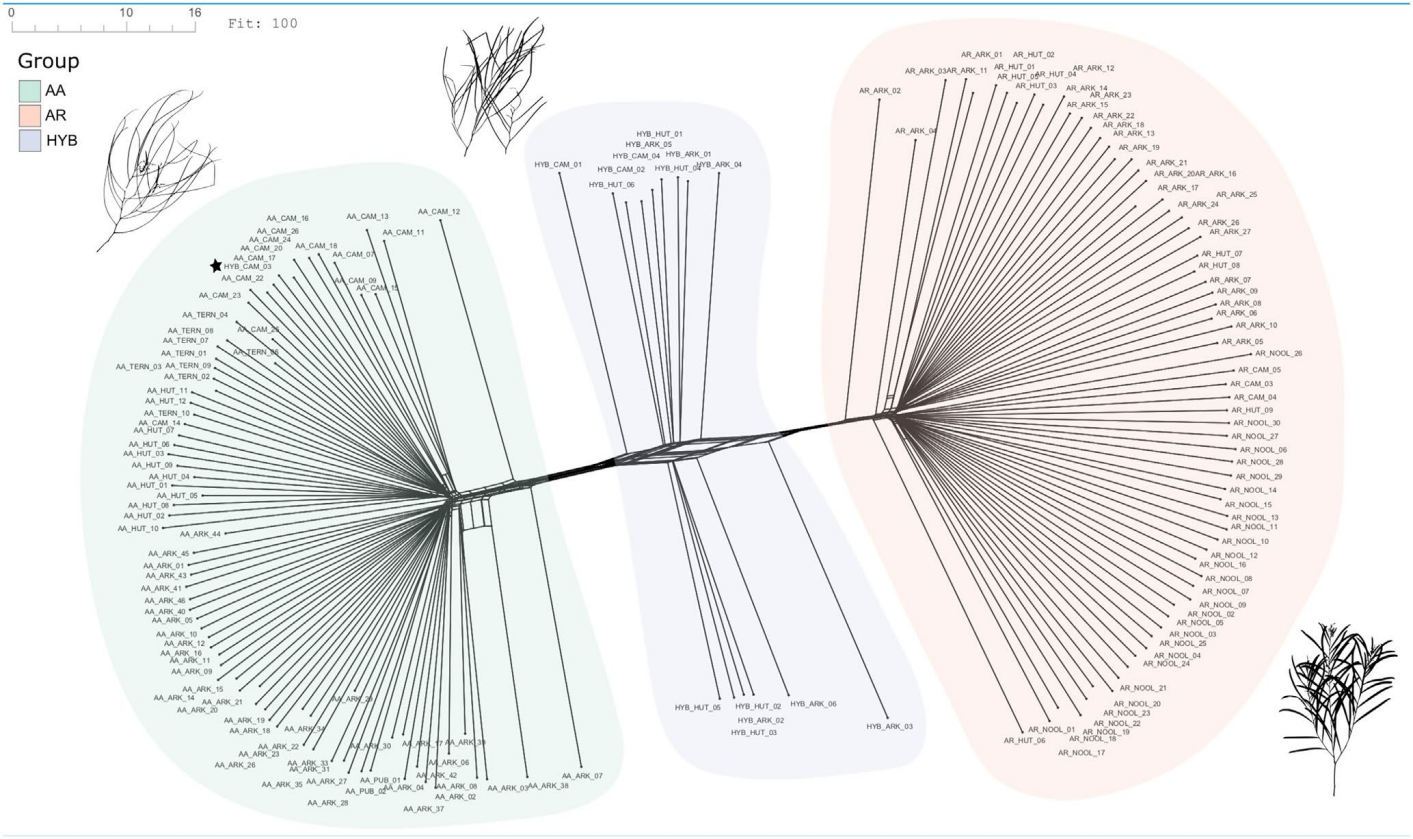
Genetic distance tree generated in SplitsTree using a distance matrix based on Euclidean distance between individuals. Here, the green shading represents *Aacacia araneosa*, purple represents putative hybrids, and orange represents *A. rivalis* . Silhouettes of individuals from each population were added to visualise phenotypic differences between groups. A single ‘*A. araneosa*’ is indicated by a black star as a possibly misidentified specimen.

We found distinct clustering by species, with putative hybrids positioned intermediate to the *A. araneosa* and *A. rivalis* clusters (Figure 4). One exception is the consistent outlier, HYB_CAM_01, which sits within the *A. araneosa* cluster, which, as above, likely represents a misidentification.

### 3.4 Isolation by Distance

Our IBD analyses using (d; *A. araneosa* only) found a significant positive correlation (*p* = 0.001) between genetic and geographic distance (Figure S1). Our *R* ^2^ indicated that 31.4% of the variation in genetic distance is explained by geographic distance—a moderately strong correlation.

### 3.5 Tess3

**FIGURE 5:**
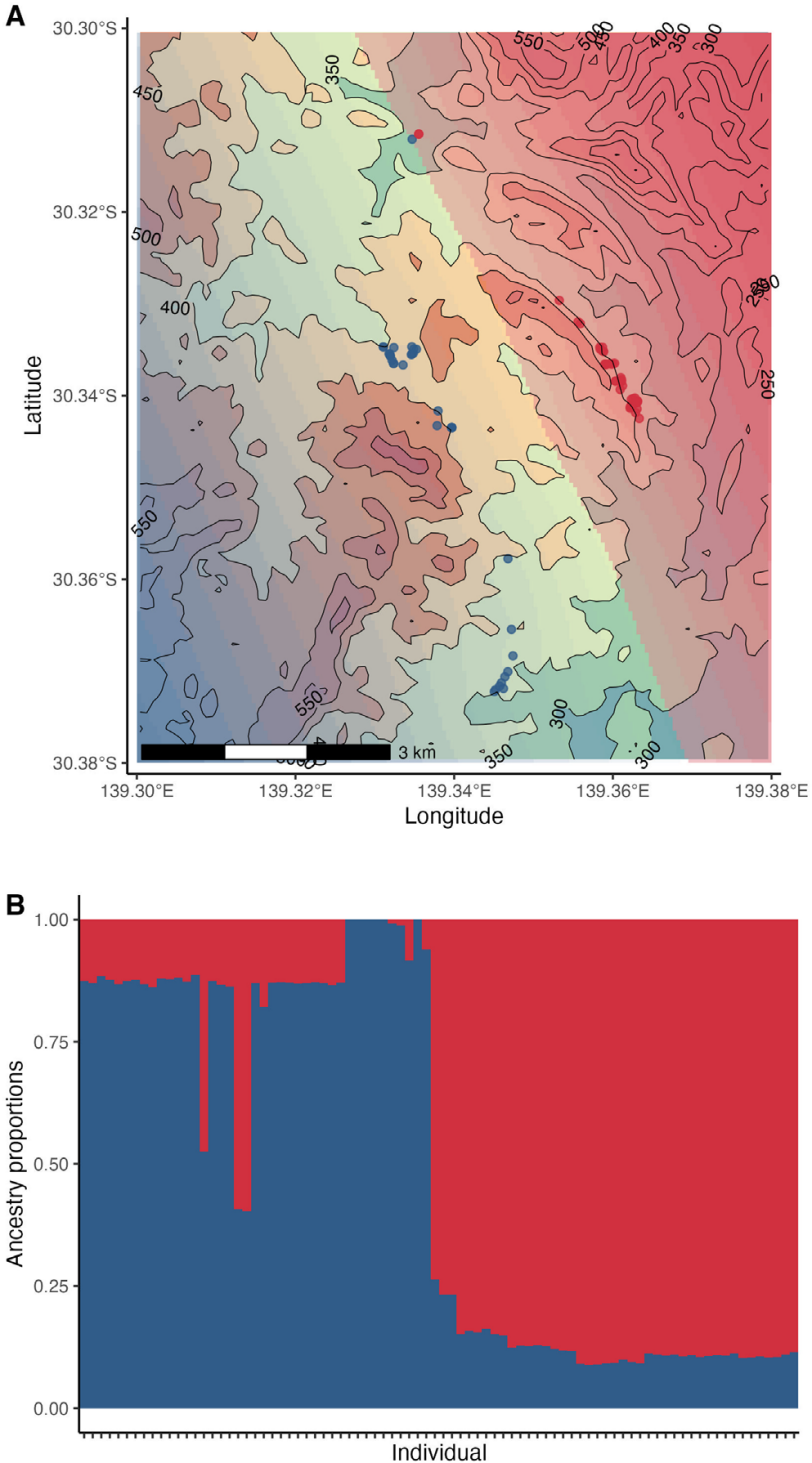
The (A) Spatial ancestry estimation interpolated using the Fields Krig Model in Tess3r (Caye et al. 2016). The points represent the sampling locations for two *Acacia araneosa* populations where colour indicates the population assignment based on (B) the ancestry proportion. Coloured isolines indicate elevation, and shaded colour indicates estimated population boundaries. The blue and red colours represent distinct genetic clusters where individuals in (B) are sorted by longitude; note that there is longitudinal overlap but not geographical overlap.

Using our (d; *A. araneosa* only) dataset, we found clear geographic structuring, with distinct genetic clusters across the *A. araneosa* range (Figure 5). Genetic discontinuity can be observed where the ridge and valley are positioned, indicating a potential barrier to gene flow (Figure 5).

### 3.6 Effective Population Size and Demographic Reconstruction

The sample sizes of the 2013 and 2018 data were insufficient to estimate the contemporary effective population size. For the 2021–22 data, we found the contemporary effective population sizes of *A. araneosa* and *A. rivalis* differed, and none of the analyses overlapped (Figure S2). For *A. araneosa*, we estimated a contemporary effective population size of 190.4 (minor allele frequency = 0.00; 95% CI = 185.3–195.7; Figure S2) and 168 (minor allele frequency = 0.05; 95% CI = 163.0–172.2; Figure S2). For *A. rivalis* , we estimated a contemporary effective population size of 79.2 (minor allele frequency = 0.00; 95% CI = 78.5–80.0; Figure S2) and 484 (minor allele frequency = 0.05; 95% CI = 459.1–511.6; Figure S2). Keeping in mind that, for *A. rivalis* , we have only sampled the small portion of its distribution which co‐occurs with *A. araneosa*.

**FIGURE 6:**
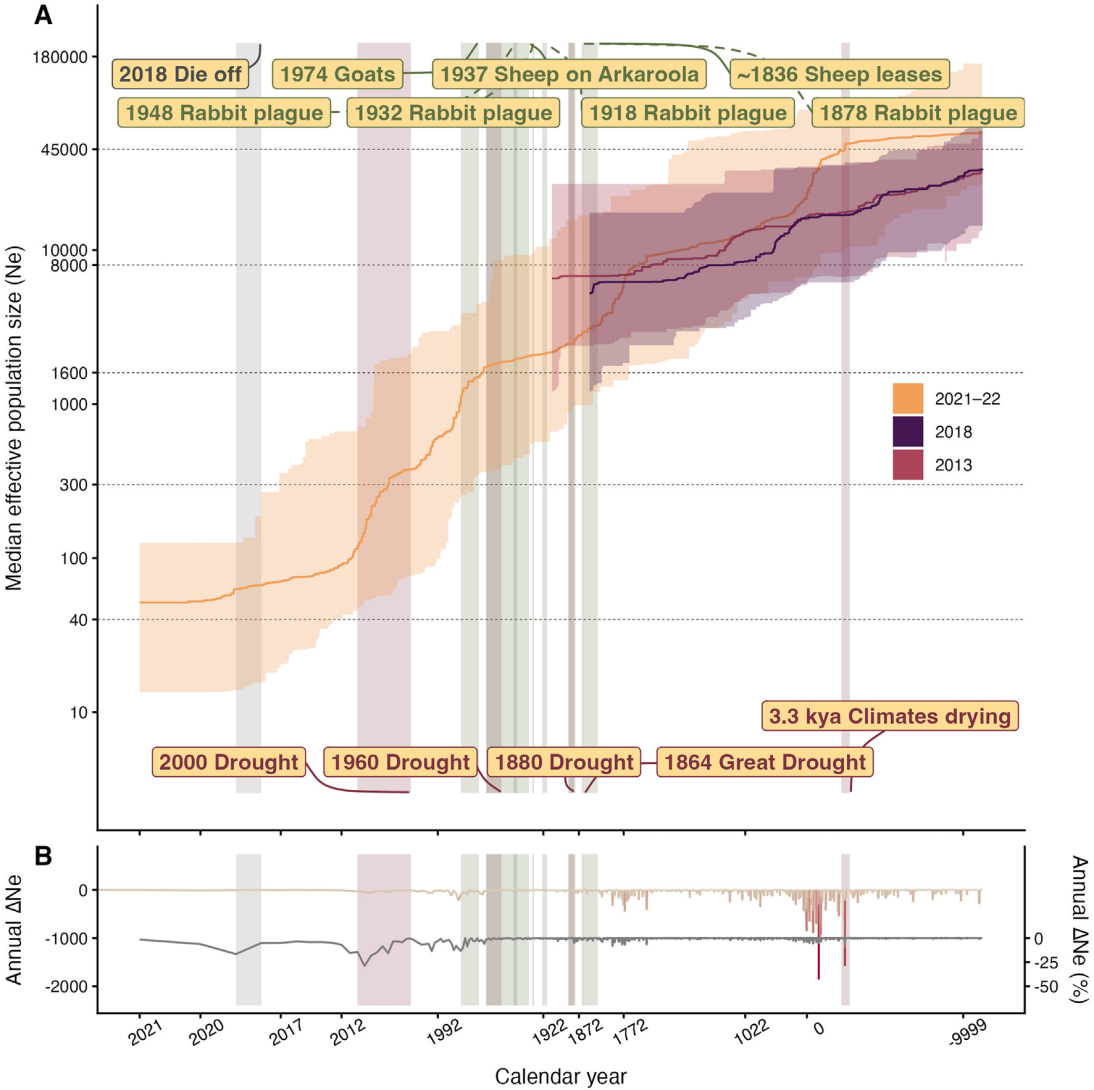
(A) Stairway plot for *Acacia araneosa* showing the median effective population size (solid lines) and 95% confidence intervals (shaded regions) using a mutation rate of 6.95 × 10^−9^ from *Arabidopsis thaliana* and 0.5 generations/year. Stairway plot colours indicate analyses restricted to the collection years of 2013 (red), 2018 (purple) and 2021–22 (orange). The main *X*‐ and *Y*‐axes are adjusted using a log10 scale. (B) The left Y‐axis shows the annual change in median effective population size (Ne) through time. The right axis and grey solid line indicate the annual change in median Ne as a percentage of the previous years' size (annual percentage change in Ne). Shaded rectangles and labels indicate known periods of potential disturbance (see Table S1) where warm rectangles indicate drought/drying events, green rectangles indicate biotic influences of pests, and the grey rectangle indicates an intrinsic decline in *A. araneosa*. Horizontal dotted lines indicate approximate effective population sizes (*Ne*) prior to declines.

Our demographic reconstruction of effective population size using Stairway Plot 2 was able to resolve a clear decline in effective population in the very recent and ancient past for *A. araneosa* for the 2021–22 dataset (Figure 6). However, the 2013 and 2018 datasets likely had insufficient sample sizes (*n* = 5 and 4, respectively) to estimate very recent change (Figure 6). Effective population size of *A. araneosa* has undergone periods of decline for about the last 3200 years, going from ~49,000 to ~51 (−99.9%) during this period (Figure 6). Since the commencement of South Australian sheep leases in ~1836 the effective population size has dropped from 3212 (Figure 6; Tables S1 and S2). However, most decline has occurred in the period prior to European settlement with the onset of climate drying 3.0–3.3 kya correlating to the start of a decline in the species (Figure 6; Tables S1 and S2). The greatest drop in effective population size occurred pre‐European settlement, with four estimated annual drops of Ne 1000–2000 around 2300 and 3100 years before present (Figure 6B; Table S2). However, because of the contemporarily very low effective population sizes, recent droughts have caused proportionally major annual losses in the remaining population. During the 2000–2010 drought there was a cumulative loss of ~74% of the effective population size in *A. araneosa* and a median 5.5% drop each year from 2011 to 2020 (Figure 6). According to the Stairway Plot 2 analysis, the contemporary median effective population size is 51, representing a drop of ~98% since 1836 (Figure 6; Table S2).

### 3.7 Kinship

First‐degree relatives were not detected among either *A. araneosa* or *A. rivalis* comparisons. One second‐degree pair (0.16%) was identified in *A. araneosa*, and two (0.08%) in *A. rivalis* . Among hybrid individuals, 66.67% of comparisons were classified as first‐degree, 16.30% as second‐degree and 17.04% as more distant (Table 1).

**TABLE 1:** Summary of pairwise kinship coefficients within groups.

Group	Pairwise comparisons (*n*)	Pairwise kinship coefficient range	Mean pairwise kinship coefficient	First‐degree (%)	Second‐degree (%)	More distant (%)
*A. araneosa*	3653	−0.48 to 0.15	−0.12	0.00	0.16	99.84
Hybrids	135.0	−0.70 to 0.28	0.14	66.67	16.30	17.04
*A. rivalis*	2414	−0.50 to 0.17	−0.12	0.00	0.08	99.92

Pairwise kinship coefficients among individuals of *A. araneosa* ranged from −0.48 to 0.15, with a mean of −0.12. For *A. rivalis* , coefficients ranged from −0.50 to 0.17, also with a mean of −0.12. Among hybrids, coefficients ranged from −0.66 to 0.28, with a mean of 0.14.

Pairwise kinship coefficients revealed contrasting patterns of relatedness within and among groups (Table 1). Kinship coefficients within the AA‐AA and AA‐AR groups were similar, ranging from −0.481 to 0.147 for AA‐AA and −0.497 to 0.173 for AR‐AR. Only a small proportion of second‐degree relatives were detected in these groups (0.16% for AA‐AA and 0.08% for AR‐AR), and no first‐degree relatives were detected.

The group kinship was markedly lower, with little evidence of recent shared ancestry. The AA‐AR comparisons ranged from −2.478 to −0.528, while AA‐HYB and AR‐HYB comparisons ranged from −1.288 to 0.083 and −1.537 to 0.038, respectively. All group pairwise comparisons were classified as more distant (< 0.088), with no first‐ or second‐degree relationships detected.

In contrast, HYB‐HYB comparisons showed substantially higher relatedness, with kinship coefficients ranging from −0.659 to 0.280. A relatively high proportion of close relatives was observed within the hybrid cohort, with 66.67% of pairwise comparisons between HYB‐HYB classified as first‐degree (≥ 0.177), 16.3% as second‐degree (≥ 0.088 and < 0.177) and 17.04% as more distant (< 0.088), suggesting high relatedness among hybrids.

### 3.8 Diversity Statistics

The results from our diversity statistics (Table 2) showed observed heterozygosity was lowest in *A. araneosa* (0.08) and highest in putative hybrids (0.19). A similar pattern was shown for both expected heterozygosity (He) and unbiased expected heterozygosity (uHe); the lowest values were shown in *A. araneosa* (0.08 and 0.08, respectively), and the highest in the putative hybrids (0.15 and 0.15, respectively). Observed heterozygosity was equal to He in *A. araneosa*, lower than He in *A. rivalis* , and higher than He in the putative hybrids. The hybrid population also exhibited a negative inbreeding coefficient (*F*_IS_ = −0.1), suggesting heterozygote excess and potential outbreeding. The fixation index (*F*_ST_) between *A. araneosa* and *A. rivalis* was found to be 0.6 (*p* = 0), indicating significant, substantial differentiation between the two species. The *F*_ST_ of the two *A. araneosa* populations was 0.04 (*p* = 0).

**TABLE 2:** *Acacia* diversity statistics calculated by locus for genlight (b; all specimens except for the outgroup). Here it is important to note that highly related individuals have been retained in the dataset, as removing first‐ and second‐degree relations, duplicate samples, or monozygotic twins from the dataset would have removed a considerable number of hybrid individuals from the analyses (see kinship results).

Group	*n*	Loci	Poly. loci	Mono. loci	Ho	He	uHe	*F*_IS_
*A. araneosa*	77	13,044	7271	5773	0.08	0.08	0.08	0.05
Hybrids	15	13,044	8477	4567	0.19	0.15	0.15	−0.10
*A. rivalis*	68	13,044	9533	3511	0.11	0.12	0.12	0.08

### 3.9 Extent of Occurrence and Area of Occupancy

For *A. araneosa*, using the Atlas of Living Australia occurrences, we calculated an extent of occurrence of 53 km^2^ and an area of occupancy of 68 km^2^. Using just our collection data, across all years, we calculated an extent of occurrence of 11 km^2^ and an area of occupancy of 24 km^2^.

### 3.10 IUCN Redlist Assessment

Under Section V of the IUCN Red List categories and criteria version 3.1, a taxon may be considered for one of the IUCN threatened categories based on population size reduction (criterion A), where it has been measured over the longer of 10 years or three generations (IUCN 2012). This decline may be observed, estimated, inferred, or suspected. Our demographic reconstruction of *A. araneosa* reveals major declines in effective population size over the past ~3000 years (99.9%) and since European colonisation (1788; 90%). While the census population size is unlikely exactly equal to the effective population size, it is a measure of an ideal population that would experience the same rate of genetic drift (Waples 2022). Hence, it is strongly relevant for conservation assessments and an ideal statistic for measuring extinction vortices (genetic drift, inbreeding and the effectiveness of selection) and should be used under Criterion A2b—inferred population size reduction using an appropriate index of abundance. The largest 10‐year drop (mostly the Millenium Drought 2003–12; 74% reduction) would qualify *A. araneosa* as Endangered.

Taxa can also be listed due to small geographic range (Criterion B) where populations are severely fragmented, have few localities, and where declines are inferred to be continuing. Our extent of occurrence and area of occupancy calculations for all historical (Atlas of Living Australia) data points were 53 and 68 km^2^, respectively (Figure S3); the former is firmly within Critically Endangered thresholds (< 100 km^2^; B1). There was a significant, but small (0.04), *F*_ST_ between the two ridgeline populations, indicating population fragmentation and *A. araneosa* has a limited number of locations (less than five; or Endangered under B2a) and continuing declines are inferred (B2b). However, from our study, the extent of occurrence and area of occupancy were smaller at 11 and 24 km^2^, respectively (Figure S3). We failed to find *A. araneosa* from localities where it has previously been found in several of the previously known grid cells (2 km^2^) that we sampled in this study (Figure S3). Additionally, many of the collections outside of this area were collected decades ago (1960–1990; Figure S3).

Considering the inferred reduction in population size and limited geographic range, our assessment is as follows:

$$\mathrm{EN}\;A2b\;\mathrm{CR}\;B1a+\text{2ab}\left(i,\mathrm{ii},v\right)$$

## 4 Discussion

Using a dataset of 13,044 filtered SNPs, we found consistent support to maintain the species concepts of both *A. araneosa* and *A. rivalis* and identified hybridisation between these species. Using a subsetted dataset of 1314 SNPs from only *A. araneosa* samples, we found evidence of strong structuring across two subpopulations, which correspond to the two remaining extant patches. However, our study also provides the first genomic analysis of *A. araneosa* and reveals that the species is quite possibly amid an ongoing 3200‐year extinction vortex, supporting a strong call to action to save the species and providing novel evidence for ancient environmental changes in arid southern Australia.

### 4.1 Species Boundaries

Our genetic analyses support the existing, and distinct, species concepts for *A. araneosa* and *A. rivalis* . Our PCoA analysis (Figure 3) shows three distinct clusters, with *A. araneosa* and *A. rivalis* clearly separated by the first principal coordinate (47.6% of variation explained), with hybrids exhibiting intermediate values. A dendrogram of genetic distance (Figure 4) also shows a similar pattern; *A. araneosa* and *A. rivalis* are placed on either end of the tree, with putative hybrids in the centre. These results suggest that hybrids are predominantly F1 hybrids with minimal backcrossing.

Our PCoA and distance tree are further supported by a fixation index (*F*_ST_) of 0.6 (*p* = 0), indicating large and significant barriers to gene flow between the two species, despite the presence of F1 hybrids. This suggests that the phenotypic differences between *A*. *araneosa* and *A. rivalis* are not due to plasticity, but rather to speciation, with little contemporary gene flow.

### 4.2 Population Structuring of *Acacia araneosa*

Within *A. araneosa*, we found strong evidence of both isolation by distance and weak population structuring (*F*_ST_ = 0.04). Our isolation by distance model indicated that geographical distance explained 31.4% of genetic variation (*p* = 0.001). Our spatial ancestry interpolation supported two populations only separated by ~2 km mostly along two east‐to‐north‐facing ridgelines (Figure 5A).

Such fine‐scale population structuring is surprising and could be explained in a combination of ways. First, seed or pollen dispersal is limited between the populations. Barriers to gene flow must exist to produce the spatial patterns that we have inferred. Second, these barriers are likely exacerbated by extremely low population sizes and an ongoing genetic bottleneck (see below), where genetic drift can quickly fix alleles in each population. In turn, this indicates both reduced adaptive capacity within populations and reduced efficiency of selection.

### 4.3 Effective Population Size, Past Demography and Ancient Environmental Change

Our estimates of contemporary effective population size (*Ne*) between both *A. araneosa* and *A. rivalis* , from NeEstimator2, were sensitive to minor allele frequency filtering (Figure S2). However, by reconstructing past demography using Stairway Plot 2, we found strong evidence that *A. araneosa* has undergone major changes in effective population size, providing an excellent demonstration of how this method can be used as a proxy when direct historical counts are unavailable. We inferred contemporary declines from an effective population size of ~4800 in 1788, before European colonisation, to 51 in 2021 (99% drop since 1788).

Most of this decline occurred between the first issued South Australian pastoral leases in 1836 and the present day. Between these dates, we inferred a 98% reduction in the median effective population size of *A. araneosa* (Figure 6). These changes are most likely attributed to both recent climatic events and post‐colonial land use activities in South Australia and the Arkaroola region. It is possible that increasing human land‐use in the area, coupled with drying and variable climate, reduced populations regionally and eventually isolated the Arkaroola populations, leading to early and dramatic drops in the inferred effective population sizes (Figure 6).

We further identified correlations between recent droughts and drops in effective population size of *A. araneosa* (Figure 6). For example, the “Great Drought” in 1864, said to have decimated the north of South Australia (Sheldrick 2010), appears to coincide with drops of 32–50 in effective population size per year (Figure 6B; Tables S1 and S2). Subsequent droughts also correspond with drops in effective population size, with a drop of ~74% in effective population size coinciding with the 2000–10 ‘Millennium Drought’ (Figure 6; Tables S1 and S2). The exact timing of declines should be interpreted with some caution as our analyses used a mutation rate from *Arabidopsis thaliana* (Weng et al. 2019) and generation times might vary.

Whilst arid‐adapted plants can be tolerant of varying climate conditions, the juvenile stages can be relatively sensitive. This has been observed in other *Acacia* species, including *Acacia lobulata* R.S.Cowan & Maslin and *Acacia verriculum* R.S.Cowan & Maslin, which are prone to high juvenile mortality during periods of drought (Buist et al. 2002). Reported observations of *A. araneosa* in the Arkaroola region during periods of drought noted high mortality and poor plant health (Doug Sprigg, pers. comm., 2021). The continued impact of severe droughts (e.g., 2000 and 2018), coupled with the ongoing increases in drought intensity more generally (Rashid and Beecham 2019) demonstrates that climatic extremes remain a major threat, even under protective land tenure.

Although Arkaroola was considered inhospitable and not stocked with sheep until 1937, feral animals were present in the area from the 1840s and experienced decades of browsing pressure by feral herbivores (feral sheep, goats, rabbits, camels and donkeys), presumably with little opportunity for recovery since seedling recruitment and adult survival are both impacted (H. Ehmann, pers. comm., 2026; D. Sprigg, pers. comm., 2026). Further, *A. araneosa* was only formally described in 1976 (Whibley 1976), supporting the notion that its range very likely extended beyond the current known distribution (Figure 2). Populations may have been lost to land use change before the species was described but are still detectable in our analysis of historical effective population size. The establishment of the Arkaroola Sanctuary in 1969, followed by the destocking of sheep from the land in 1970, was closely followed by a brief, drastic increase in goat numbers in 1974, which also aligns with a visible reduction in effective population size (Figure 6). Davies (1995) found evidence of extensive browsing by goats which resulted in high mortality rates of adults in *A. araneosa*, suggesting that our analysis detected the impact of increased goat browsing. A major goat eradication programme (1968–1983, ~96,000 removed) likely eased grazing pressure. However, following wet years between 1987 and 1992, when many seedlings were observed, goats were blamed for their disappearance and overall poor recruitment (Davies 1995). Additionally, intense goat grazing near permanent waterholes on *A. araneosa*'s southern limits (Davies 1990) might explain the lack of recent southern records (Figure S3) and, in part, the isolation between the two populations. Four rabbit plagues are known from the region and have also been blamed for poor seedling recruitment (H. Ehmann, pers. comm., 2026; Figure 6A; Tables S1 and S2). This demonstrates how pests and climate can interact to deny population recovery even in good years.

We inferred ancient effective population sizes drops from ~49,000 (3.3 kya) to ~4800 (1788) before European colonisation (90% drop; 99.9% until 2021). From 3.0–3.3 kya, Australia's arid interior is thought to have experienced an intensified drying period (Donders et al. 2007)—which has also been linked with the extinction of mainland thylacines (White et al. 2017). The timing of this event coincides with the start of the greatest decline in *A. araneosa*'s effective population size (90% drop ~3.3 kya until 1788; Figure 6). That contemporary declines are also associated with droughts, and likely exacerbated by invasive herbivores, provides further evidence for *A. araneosa*'s sensitivity to drier climates and support for ancient drying in the region.

The period 2–5 kya in Australia was generally characterised by increasingly variable and drier conditions, with greater El Niño frequency, increased fire and extended droughts (Reeves et al. 2013), however, paleoclimatic records up until this point are sparse and uncertain. We suggest that the observed drops in effective population size during this time (a) occurred across a much broader area of arid southern Australia than the contemporary distribution of *A. araneosa* (Figure 2) and (b) were driven by late Holocene droughts. We hypothesise that this signal will be detectable—as either effective population size increases or decreases—in other sensitive species across the region.

### 4.4 Genetic Diversity and Kinship

We found that *A. araneosa* displayed the lowest genetic diversity among the groups (*A. araneosa*, *A. rivalis* and hybrids) and showed a moderately positive inbreeding coefficient. However, kinship analyses indicated low relatedness among *A. araneosa* individuals, with fewer than 2% of pairwise comparisons classified as second‐degree relatives. This finding should be interpreted cautiously, as sampling efforts were designed to avoid nearest neighbours where possible and minimise inclusion of close relatives. Nevertheless, similarly low levels of kinship in conjunction with low genetic diversity have been observed in other threatened Australian *Acacia* species with fragmented distributions and restricted ranges (Blyth et al. 2020). Most Australian *Acacia* species outcross, and experimental studies have detected inbreeding avoidance mechanisms following mating among close relatives (Kenrick et al. 1987; Kenrick and Knox 1989). In *Acacia whibleyana* R.S.Cowan & Maslin, for example, low seed set was hypothesised to result from inbreeding avoidance mechanisms, supported by unexpectedly low kinship estimates and limited genetic diversity (Blyth et al. 2020). Here, there is a substantial risk that reduced mate availability, combined with limited dispersal and pollen transfer, will increase inbreeding depression through genetic drift. This risk may be further compounded by reduced reproductive success arising from kin recognition and inbreeding avoidance mechanisms, thereby accelerating progression towards an extinction vortex.

The hybrid group exhibited the highest observed heterozygosity, low *F*_IS_, and consistently high pairwise kinship, indicating that they originate from a narrow set of hybridisation events—likely a single cross or a small number of related founders. Again, caution is required when comparing this result to *A*. *araneosa*, as all hybrids, but not all *A. araneosa* or *A. rivalis*, were sampled, potentially retaining a higher number of siblings in the dataset. While hybridisation can pose a risk to threatened species in the form of genetic swamping, in this instance, due to minimal observed backcrossing, we believe the threat hybridisation with *A. rivalis* and *A. araneosa* to be low.

### 4.5 Threatened Species Status

*Acacia araneosa* is listed as Endangered under the South Australian National Parks and Wildlife Act of 1972 and as Vulnerable under the national Environmental Protection and Biodiversity Conservation (EPBC) Act of 1999. As of October 2025, the International Union for Conservation of Nature and Natural Resources (IUCN) lists 27 *Acacia* species as Vulnerable (12), Endangered (13) and Critically Endangered (two). Considering the ongoing stressors acting on *A. araneosa*, we suggest that it is eligible for updated listing under the EPBC and novel listing under the IUCN.

Using IUCN criteria, we assessed *A. araneosa* as eligible to be listed as Endangered—under A2b—and Critically Endangered—under B1a + 2ab(i,ii,v). *Acacia araneosa* approaches 80% threshold for Critically Endangered under A2b, with a 74% reduction during the 10‐year period encompassing the Millenium Drought (2003–12). Populations declines have been long‐term, ongoing and stochastically severe (Figure 6). *Acacia araneosa* exceeds the < 100 km^2^ (B1) area of occupancy threshold for Critically Endangered with the most‐conservative estimate of 68 km^2^ (including historical records) and the less‐conservative estimate of 24 km^2^ (this study alone). *Acacia araneosa* also has few known localities and shows signs of population fragmentation, as indicated by the results of our spatial ancestry estimations (Figure 5) and an *F*_ST_ of 0.04 (*p* = 0) between populations. We highlight that *A. araneosa* is susceptible to demographic and environmental stochasticity, has been impacted by massive past declines and ongoing genetic drift, and has reduced adaptive capacities. Hence, we strongly recommend that *A. araneosa* is listed as Critically Endangered—EN A2b CR B1a + 2ab(i,ii,v)—under both the IUCN and EPBC and that monitoring, expanded sampling regimes and immediate conservation actions are required.

## 5 Conclusion

Our study supports the existing species concepts of *A. araneosa* and *A. rivalis* . While these species hybridise, there was no evidence of F2 individuals, and hybridisation does not appear to be a process that poses a major threat to the persistence of either species.

However, *A. araneosa* exhibited significant genetic isolation by distance, population structuring, low genetic diversity, inbreeding, a small (and likely reduced) geographic range, and large historical and contemporary reductions in effective population size. The timing of contemporary and ancient declines in effective population size coincided well with drying events and feral goats. We hypothesise that population recovery has been denied during good years due to feral herbivores. This provides novel evidence for continued drying in arid southern Australia where paleoclimatic data are scattered and uncertain.

We highlight the importance of using demographic reconstructions as a powerful tool to uncover contemporary and ancient population trends that are essentially impossible using standard ecological methods. We also demonstrate how we can use empirical genetic data to build a holistic picture of conservation issues that pre‐dates and contextualises the impacts of European colonisation. Taken together, these can justify a Critically Endangered species listing under IUCN criteria and provide the insights needed for greater targeted future work into *A. araneosa*, or other threatened taxa.

## Author Contributions

**Brittany P. Hogben:** conceptualization (equal), data curation (equal), formal analysis (equal), investigation (equal), methodology (equal), visualization (equal), writing – original draft (equal), writing – review and editing (equal). **Andrew J. Lowe:** conceptualization (equal), funding acquisition (equal), writing – original draft (equal), writing – review and editing (equal). **Chelsea Hampel:** data curation (equal), investigation (equal), methodology (equal), writing – original draft (equal). **Lars Littmann:** data curation (equal), formal analysis (equal), investigation (equal). **Martin O'Leary:** data curation (equal), formal analysis (equal), investigation (equal). **James B. Dorey:** data curation (equal), formal analysis (equal), investigation (equal), methodology (equal), supervision (equal), validation (equal), visualization (equal), writing – original draft (equal), writing – review and editing (equal). **Colette Blyth:** conceptualization (equal), data curation (equal), formal analysis (equal), funding acquisition (equal), investigation (equal), methodology (equal), project administration (equal), resources (equal), supervision (equal), validation (equal), visualization (equal), writing – original draft (equal), writing – review and editing (equal).

## Funding

All library preparation and sequencing costs were funded by the Threatened Species Initiative. The Threatened Species Initiative is supported by funding from Bioplatforms Australia through the Australian Government National Collaborative Research Infrastructure Strategy (NCRIS), in partnership with the University of Sydney, Australian Government Department of Environment and Energy, WA Department of Biodiversity, Conservation & Attractions, Amazon Web Services, NSW Saving Our Species, Australian Wildlife Conservancy and the Zoo and Aquarium Association. We would like to acknowledge the contribution of the Threatened Species Initiative consortium in the generation of data used in this publication. We would also like to acknowledge that this project received support through the Terrestrial Ecosystem Research Network (TERN) infrastructure, which is also enabled by the Australian Government's National Collaborative Research Infrastructure Strategy (NCRIS).

## Disclosure

Collection permit details: Class D (95/0915‐2022D). Class A (95/0916_2022). Class D (95/0505‐D_2021). Y26200‐8 (Permit to Undertake Scientific Research). Y26200‐1 (Permit to Undertake Scientific Research).

## Conflicts of Interest

The authors declare no conflicts of interest.

## Supporting information


**Figure S1:** Isolation by distance (IBD) analysis on genlight (d; *Acacia araneosa* only) calculated using Euclidean distance.
**Figure S2:** The estimated contemporary effective population sizes (Ne) of *Acacia araneosa* and *A. rivalis* using the minor allele frequencies 0.00 (sand) and 0.05 (green). Samples are from the 2021–22 datasets.
**Figure S3:** Map showing the geographic extent of *Acacia araneosa* with (blue points, minimum‐spanning polygon and 2 km^2^ grids) *A. araneosa* collected in our study, (yellow points, minimum‐spanning polygon and 2 km^2^ grids) *A. araneosa* from the Atlas of Living Australia, and (black crosses) *A. rivalis* or hybrid samples from our current study where no *A. araneosa* where found. The fill colours for *A. araneosa* from the Atlas of Living Australia show the year of collection where older collections are darker and newer are lighter.

**Table S1:** demographic notes of some potential impacts on *Acacia araneosa* and their sources.

**Table S2:** For *Acacia araneosa*, the year before present, where zero = 2022, median drop in effective population size (Ne), actual drop (compared to the year prior), the rate of change drop (proportion and percentage), the calendar year of the drop and the proportion drop over the previous 10 years (IUCN criterion).

## Data Availability

The data that support the findings of this study are openly available in GitHub at https://github.com/jbdorey/Acacia_SNPs and Figshare at https://doi.org/10.25909/32646513.
