# Supplementary material for: Population Collapse of the Spidery Wattle (Acacia araneosa) During Late Holocene Aridification: Genomic Evidence for Critically Endangered Status
Source: Ecol Evol. 2026 Jul 10;16(7):e73959. doi: 10.1002/ece3.73959 (PMC13354547; doi:10.1002/ece3.73959)
Supplement: Supplementary file 1 — Figure S1: Isolation by distance (IBD) analysis on genlight (d; Acacia araneosa only) calculated using Euclidean distance. Figure S2: The estimated contemporary effective population sizes (Ne) of Acacia araneosa and A. rivalis using the minor allele frequencies 0.00 (sand) and 0.05 (green). Samples are from the 2021–22 datasets. Figure S3: Map showing the geographic extent of Acacia araneosa with (blue points, minimum‐spanning polygon and 2 km2 grids) A. araneosa collected in our study, (yellow points, minimum‐spanning polygon and 2 km2 grids) A. araneosa from the Atlas of Living Australia, and (black crosses) A. rivalis or hybrid samples from our current study where no A. araneosa where found. The fill colours for A. araneosa from the Atlas of Living Australia show the year of collection where older collections are darker and newer are lighter. [file ECE3-16-e73959-s002.docx]

Supplementary Material

**Figures**


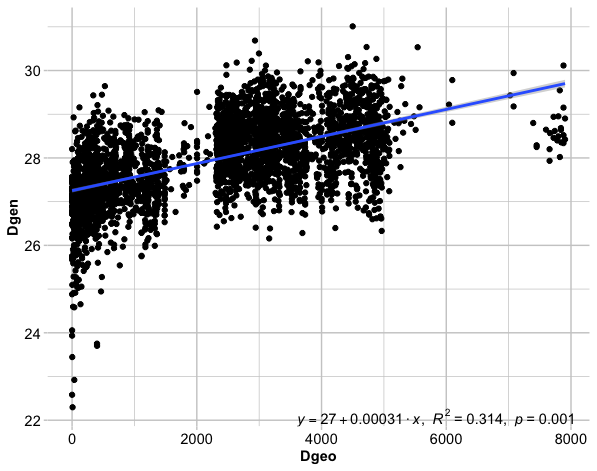


**Figure S1.** Isolation by distance (IBD) analysis on genlight (d; *Acacia araneosa* only) calculated using Euclidean distance.


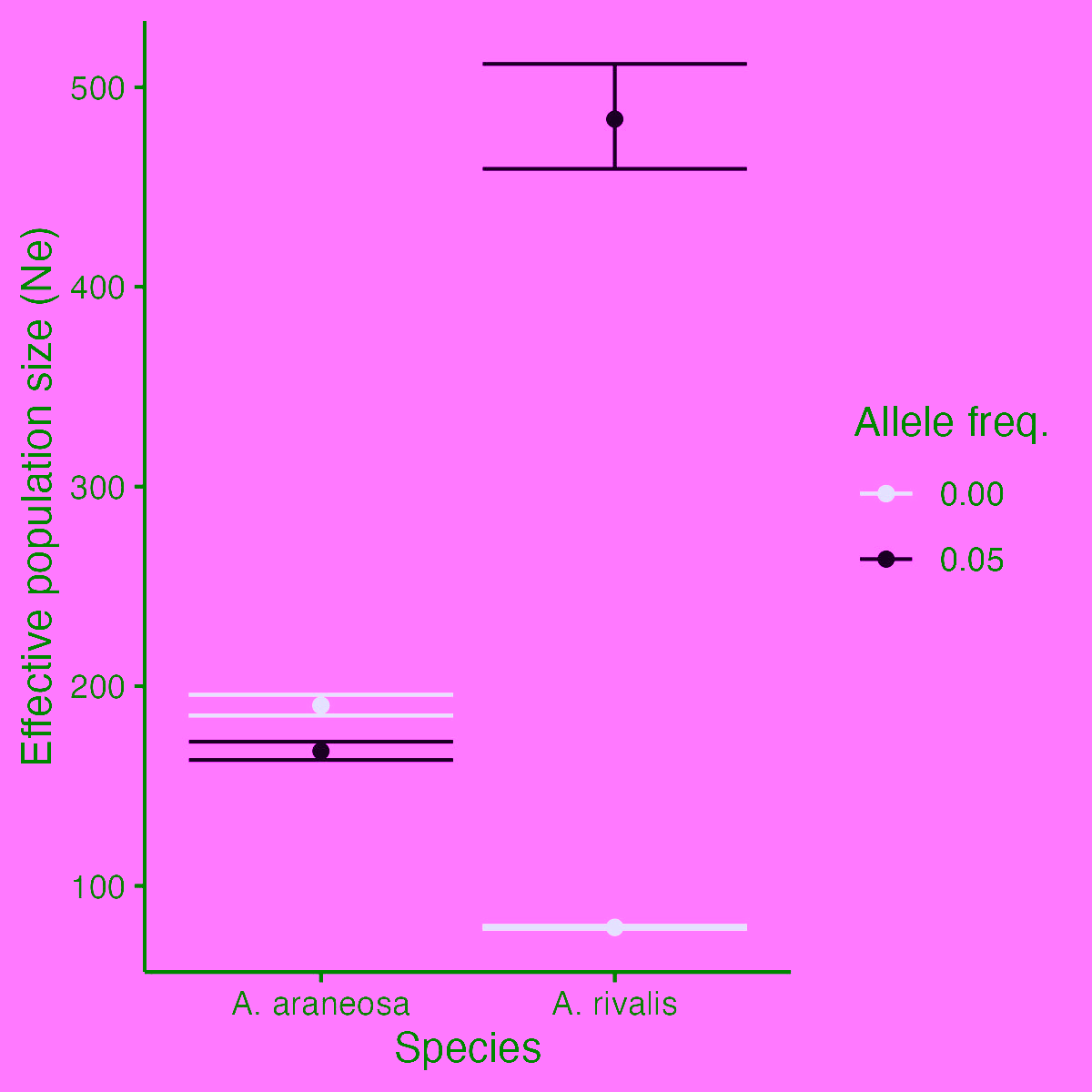


**Figure S2.** The estimated contemporary effective population sizes (Ne) of *Acacia araneosa* and *A. rivalis* using the minor allele frequencies 0.00 (sand) and 0.05 (green). Samples are from the 2021–22 datasets.

**Figure S3**. Map showing the geographic extent of *Acacia araneosa* with (blue points, minimum-spanning polygon, and 2 km^2^ grids) *A. araneosa* collected in our study, (yellow points, minimum-spanning polygon, and 2 km^2^ grids) *A. araneosa* from the Atlas of Living Australia, and (black crosses) *A. rivalis* or hybrid samples from our current study where no *A. araneosa* where found. The fill colours for *A. araneosa* from the Atlas of Living Australia show the year of collection where older collections are darker and newer are lighter.

**Tables (please refer to separate excel file)**

**Table S1**. demographic notes of some potential impacts on *Acacia araneosa* and their sources.

**Table S2.** For *Acacia araneosa*, the year before present, where zero = 2022, median drop in effective population size (*Ne*), actual drop (compared to the year prior), the rate of change drop (proportion and percentage), the calendar year of the drop, and the proportion drop over the previous ten years (IUCN criterion).
